# Data demonstrating the effects of build orientation and heat treatment on fatigue behavior of selective laser melted 17–4 PH stainless steel

**DOI:** 10.1016/j.dib.2016.02.013

**Published:** 2016-02-10

**Authors:** Aref Yadollahi, Jutima Simsiriwong, Scott M. Thompson, Nima Shamsaei

**Affiliations:** a Department of Mechanical Engineering, Mississippi State University, Box 9552, Mississippi State, MS 39762, USA; b Center for Advanced Vehicular Systems (CAVS), Mississippi State University, Box 5405, Mississippi State, MS 39762, USA

**Keywords:** Additive manufacturing (AM), Fatigue, Cyclic deformation, Cyclic stress–strain responses, Fractography

## Abstract

Axial fully-reversed strain-controlled (R=−1) fatigue experiments were performed to obtain data demonstrating the effects of building orientation (i.e. vertical versus horizontal) and heat treatment on the fatigue behavior of 17–4 PH stainless steel (SS) fabricated via Selective Laser Melting (SLM) (Yadollahi et al., submitted for publication [1]). This data article provides detailed experimental data including cyclic stress-strain responses, variations of peak stresses during cyclic deformation, and fractography of post-mortem specimens for SLM 17–4 PH SS.

Specifications Table

**Table:** 

Subject area	Mechanical and Material Science Engineering
More specific subject area	Fatigue, Additive Manufacturing
Type of data	Table (Microsoft Excel file format), Microscopy images
How data was acquired	Strain-controlled fatigue experiments (laboratory), Scanning electron microscopy (SEM)
Data format	Raw and analyzed/processed
Experimental factors	Test specimens were fabricated in vertical and horizontal directions via the Selective Laser Melting (SLM) technique. Heat treatments (solution annealing and aging) were applied to some of the as-built specimens. Axial fatigue experiments were conducted following ASTM International guidelines as appropriate.
Experimental features	Effects of building orientation and heat treatment on fatigue behavior of SLM 17–4 PH stainless steel specimens are demonstrated via processed data, while fractography of post mortem specimens was performed via microscopy to provide raw images of fracture surfaces.
Data source location	Center for Advanced Vehicular Systems (CAVS), Mississippi State University, Mississippi State, MS, USA
Data accessibility	Data is within this article.

## Value of the data

• The research data provided herein can be used to demonstrate the effects of building orientation and heat treatment on fatigue behavior of 17–4 PH stainless steel (SS) fabricated via Selective Laser Melting (SLM) process. The data allows for the comparison of fatigue properties of 17–4 PH SS parts manufactured via SLM technique with those of conventionally-manufactured parts.
• Images obtained via fractography may be used to determine the crack initiation and failure mechanisms within additively manufactured parts during cyclic loading.
• The presented data may be used for determining methods for improving the quality of components fabricated via SLM.
• The data presented herein may be used to develop microstructure-sensitive fatigue models, or plasticity models, for more accurate prediction of fatigue life or deformation behavior under cyclic loading, respectively.

## 1 Data

Provided data were obtained from performing axial fully-reversed, strain-controlled fatigue experiments on 17–4 PH SS specimens fabricated via SLM. Building orientation of specimens as well as their post-SLM heat treatments were varied and are summarized in Table 1. The data included in this article are based on experimental results provided in previous publications from the authors [1]. All data have been deposited to the Data in Brief Dataverse: 10.7910/DVN/THM2DN.

## 2 Experimental design, materials, and methods

In order to investigate the effect of building orientation, two different sets of 17–4 PH SS samples were fabricated using gas-atomized 17–4 PH SS powder via a ProX™ 100 SLM system. The first set contained ‘vertically-deposited’ cylindrical rods, with 8 mm diameter and 75 mm height, while the second set contained ‘horizontally-orientated’ pseudo-cylinders. Samples were fabricated on a non-preheated build plate. Process parameters (i.e. laser power, scanning speed, layer thickness, and hatching pitch) were optimized to obtain an acceptable level of final part density using a design of experiments methodology. Selected process parameters: laser power of 48 W, traverse speed of 300 mm/s, layer thickness of 30 mm, and hatching pitch of 50 mm, were fixed during fabrication of both specimen sets. Samples were not thermally stress relieved prior to their removal (cutting) from the build plate. Material used as support structure during fabrication of horizontal samples was removed using a lathe. In order to investigate the effect of heat treatment, half of the as-built (AB) samples, from both the vertical and horizontal sets, underwent solution annealing (Condition A) and then peak-aging (Condition H900). In the end, four sets of samples were generated for characterization, namely: vertical samples in the AB and heat treated (HT) conditions as well as horizontal samples in AB and HT conditions. All samples were machined into round fatigue specimens with uniform gage section (4 mm gage diameter, 18 mm gage length, 30 mm fillet radius, 7 mm grip diameter, and 22.5 mm grip length) following the ASTM E606 standard [2]. After machining, the gage sections of fatigue specimens were polished to a roughness below ~0.7 μm. Axial fatigue experiments were conducted in accordance with ASTM E606 [2] at room temperature under a sinusoidal loading waveform until failure occurred or the number of cycles, *N*_f_, reached 10^6^ (i.e. run-out). Fatigue test frequencies were adjusted based on the test strain amplitudes, as presented in Table 1, so that strain rate was approximately the same for all fatigue tests. Fatigue fracture surfaces were examined using scanning electron microscopy (SEM) to determine the sites of fracture initiation and failure mechanisms. More details of experimental setup and results can be found in [1].

## Figures and Tables

**Table 1:** Summary of axial fully-reversed (R=−1) strain-controlled fatigue tests.

Specimen ID	Strain Amplitude, $\varepsilon_{a}$ (%)	Frequency (Hz)	Reversals to Failure, $2N_{f}$
*Vertical AB*			
01	0.50	0.5	248
02	0.40	1.0	1,872
03	0.37	1.0	1,544
04	0.30	1.5	7,696
05	0.30	1.5	12,844
06	0.20	3.0	125,438
07	0.20	3.0	179,122
08	0.15	5.0	366,504
09	0.15	5.0	473,640
10	0.15	5.0	>2,486,768

*Vertical HT*			
01	0.50	0.5	824
02	0.40	1.0	3,708
03	0.40	1.0	5,748
04	0.30	1.5	20,178
05	0.30	1.5	23,400
06	0.20	3.0	96,150
07	0.20	3.0	100,384
08	0.15	5.0	75,564
09	0.15	5.0	331,278
10	0.12	5.0	204,086
11	0.12	5.0	632,756
12	0.10	7.0	840,624

*Horizontal AB*			
01	0.50	0.5	930
02	0.40	1.0	3,330
03	0.40	1.0	4,688
04	0.30	1.5	18,426
05	0.30	1.5	29,050
06	0.20	3.0	186,940
07	0.20	3.0	234,942
08	0.20	3.0	564,464
09	0.20	3.0	1,103,298
10	0.18	4.0	>2,235,036

*Horizontal HT*			
01	0.50	0.5	1,926
02	0.40	1.0	8,352
03	0.40	1.0	11,422
04	0.30	1.5	28,560
05	0.30	1.5	30,750
06	0.25	3.0	51,782
07	0.25	3.0	94,506
08	0.20	3.0	321,142
09	0.18	4.0	283,626
10	0.18	4.0	198,472
11	0.15	5.0	222,200
12	0.12	5.0	666,912
13	0.10	7.0	1,219,466
