# Supplementary material for: Data demonstrating the effects of build orientation and heat treatment on fatigue behavior of selective laser melted 17–4 PH stainless steel
Source: Data Brief. 2016 Feb 10;7:89–92. doi: 10.1016/j.dib.2016.02.013 (PMC4761700; doi:10.1016/j.dib.2016.02.013)
Supplement: Supplementary file 2 — Supplementary material [file mmc2.pdf]

Appendix A: Supporting Information

All of the raw fatigue data as well as fractography images obtained via SEM of fatigued SLM 17-4 PH SS specimens exist within the Data in Brief Dataverse. This dataset includes the following directories:

- Cyclic Hysteresis Acquisition contains all cyclic stress-strain responses (hysteresis loops) of the fully-reversed strain-controlled tests at different strain amplitudes for all conditions in [1]. When the file is unzipped, four Microsoft Excel® files can be found in a folder labeled "Cyclic Hysteresis Acquisition", corresponding to: the vertical set in AB condition, horizontal set in AB condition, vertical set in HT condition, and horizontal set in HT condition. Each Excel file contains the segments (which is equivalent to the number of reversals) and hysteresis stress-strain responses for each specimen, as depicted in Table 1, in a separate worksheet (tabs found at bottom of screen in Excel).
- Peak Valley contains data used to generate variations of tensile and compressive peak stresses during cyclic deformation at different strain amplitudes for all conditions in [1]. When the file is unzipped, four Microsoft Excel® files can be found in a folder labeled "Peak Valley", corresponding to the: vertical set in AB condition, horizontal set in AB condition, vertical set in HT condition, and horizontal set in HT condition. Each Excel file contains the peak stress versus segment (reversals) for each specimen, as listed in Table 1, in a separate worksheet.
- Fatigue Fractography contains the SEM fractography images of failed (post-mortem) specimens. Images were organized according to 'specimen ID' as presented in Table 1. When the file is unzipped, four folders can be found, corresponding to the: vertical set in AB

condition, horizontal set in AB condition, vertical set in HT condition, and horizontal set in HT condition. Images are in the TIF format with a size of 640×480 pixels.
